# The role of factor V in trauma-induced coagulopathy: an observational and experimental study

**DOI:** 10.1016/j.rpth.2025.102857

**Published:** 2025-04-17

**Authors:** Pieter H. Sloos, Romein W.G. Dujardin, Joost C.M. Meijers, Christine Gaarder, Ross Davenport, Simon Stanworth, Pär I. Johansson, Jakob Stensballe, Marc Maegele, Nicole P. Juffermans, Derek J.B. Kleinveld

**Affiliations:** 1 Department of Intensive Care Medicine, Amsterdam University Medical Center location University of Amsterdam, Amsterdam, the Netherlands; 2 Amsterdam Institute for Infection and Immunity, Laboratory of Experimental Intensive Care and Anesthesiology, Amsterdam University Medical Center location University of Amsterdam, Amsterdam, the Netherlands; 3 Department of Anesthesiology, Amsterdam University Medical Center location University of Amsterdam, Amsterdam, the Netherlands; 4 Department of Experimental Vascular Medicine, Amsterdam University Medical Center location University of Amsterdam, Amsterdam, the Netherlands; 5 Amsterdam Cardiovascular Sciences, Pulmonary Hypertension and Thrombosis, Amsterdam, the Netherlands; 6 Department of Traumatology, Oslo University Hospital, Oslo, Norway; 7 Department of Trauma Surgery, Barts and the London School of Medicine and Dentistry, London, United Kingdom; 8 National Health Service Blood and Transplant/Oxford University Hospital NHS Trust, John Radcliffe Hospital, Oxford, United Kingdom; 9 Department of Anesthesiology and Trauma Center, Center for Head and Orthopedics, Section for Transfusion Medicine, Copenhagen University Hospital Rigshospitalet, Capital Region Blood Bank, Copenhagen, Denmark; 10 Department of Traumatology and Orthopedic Surgery, Cologne–Merheim Medical Center, University of Witten/Herdecke, Cologne, Germany; 11 Department of Intensive Care Medicine, Erasmus Medical Center, Rotterdam, the Netherlands; 12 Department of Anesthesiology, Erasmus Medical Center, Rotterdam, the Netherlands

**Keywords:** hemostasis, blood coagulation factors, factor V, wounds and Injuries, hemorrhage

## Abstract

**Background:**

In bleeding patients with trauma-induced coagulopathy (TIC), factor (F)V becomes depleted, which may not be corrected with existing treatment strategies. It is currently unknown whether supplementing FV or FVa improves TIC.

**Objectives:**

To explore the relationship between FV activity and mortality in trauma patients, and to investigate the effect of FV(a) supplementation in addition to other treatment strategies in an *in vitro* model of TIC.

**Methods:**

The association between FV activity and mortality was studied using an international prospective cohort study of trauma patients. In an *in vitro* whole blood and plasma model of TIC, the effect of FV(a) on rotational thromboelastometry and fibrin formation was studied. Effects of FV(a) were evaluated either as a standalone therapy or as adjunctive therapy in combination with tranexamic acid, fibrinogen concentrate, and/or prothrombin complex concentrate.

**Results:**

A total of 1285 patients were included, with a median injury severity score of 16 (interquartile range: 8-26). Decreased FV activity was associated with increased mortality. In the whole blood TIC model, FVa increased maximum clot firmness and reduced fibrinolysis, both as a single and combination therapy. In the plasma TIC model, with lower tissue factor concentrations than in the whole blood model, FV(a) prolonged clotting times, both as a single treatment and in combination with other treatments.

**Conclusion:**

FV depletion after trauma is associated with increased mortality. In an *in vitro* model of TIC, FV(a) results in procoagulant, antifibrinolytic, and anticoagulant effects. Further research is highly warranted.

## Essentials

• Depletion of coagulation FV may contribute to trauma-induced coagulopathy (TIC).
• FV activity was assessed in trauma patients. The effect of FV on TIC was studied *in vitro.*
• Reduced FV activity was associated with increased mortality after trauma.
• *In vitro*, FV results in procoagulant, antifibrinolytic, and anticoagulant effects.

## 1 Introduction

Trauma-induced coagulopathy (TIC) is a complex and life-threatening condition that affects around 25% of bleeding trauma patients [1]. TIC is characterized by platelet dysfunction, coagulation factor deficiency, and hyperfibrinolysis [1,2]. The current treatment strategy consists of restrictive amounts of crystalloid infusion, early tranexamic acid (TXA), and blood transfusion in a balanced ratio of blood components [[3], [4], [5]]. Recent randomized clinical trials failed to show a benefit of empiric therapy with either fibrinogen or prothrombin complex concentrate (PCC) [6,7]. Therefore, an urgent need for novel treatment options for TIC remains.

Coagulation factor deficiency is a defining feature of TIC, but not all factors are depleted to the same extent, and some remain within their normal range in patients with TIC [8,9]. Factor (F)V is one of the most severely depleted coagulation factors early after injury, correlating with TIC as assessed by rotational thromboelastometry (ROTEM) [8,10]. The early depletion of FV activity after trauma may be a result of excessive activation of coagulation or degradation by activated protein C (aPC) and plasmin [11,12].

FV itself has no procoagulant activity. However, once FV is activated by thrombin or FXa into FVa, it accelerates the conversion of FX to FXa by several orders of magnitude, thereby amplifying thrombin formation [13,14]. On the other hand, FV has several anticoagulant functions mediated through its association with the aPC and protein S (PS) complex and through tissue factor (TF) pathway inhibitor [15,16]. It is currently unknown whether restoring low FV activity with purified FV or FVa could improve TIC.

In this study, we aimed to determine the association of diminished FV activity with mortality after trauma. Using an *in vitro* model of TIC, we aimed to unravel the effect of purified FV(a) in addition to TXA, fibrinogen concentrate, and PCC on coagulopathy outcome markers. We hypothesize that early after trauma and decreased FV activity is associated with increased mortality and supplementation of FV(a) improves clot formation and clot strength in an *in vitro* model of TIC.

## 2 Methods

### 2.1 Trauma population

A post hoc analysis was conducted on the prospective cohort study “Activation of Coagulation and Inflammation in Trauma III” trial, which included patients after full trauma team activation in 6 European level 1 trauma centers in Amsterdam, the Netherlands; Cologne, Germany; Copenhagen, Denmark; London, the United Kingdom; Oslo, Norway; and Oxford, the United Kingdom. Patients were included based on clinical signs of severe injury and/or shock. Patients were excluded if they presented >120 minutes after injury, were referred from another hospital, or received >2000 mL of intravenous fluids before arriving at the hospital. A complete list of inclusion and exclusion criteria can be found in Supplementary Table S1. For the present analysis, patients were included between January 2008 and November 2016.

The study was performed in accordance with the Declaration of Helsinki and after approval by East London and City Research Ethics Committee (07/Q0603/29) and the national ethics committees of all participating centers. After initial deferred consent, informed consent was obtained from all patients or their relatives within 24 hours after inclusion.

Blood was collected from patients upon arrival to the emergency department within 2 hours after traumatic injury. Citrated blood was centrifuged twice at 1750 × *g* for 10 minutes, and plasma was stored at −80 °C for later analysis. Patient and injury characteristics were recorded in a central database. FV activity was measured using the Sysmex CA-CS2100i analyzer (Siemens), based on which patients were stratified into quartiles. In-hospital mortality due to any cause was recorded as mortality at 12 hours and mortality at 28 days.

### 2.2 *In vitro* TIC model

Venous blood was withdrawn from healthy male volunteers after institutional ethical approval (NL82402.018.22) and prior informed consent from the volunteers. Only male volunteers were included to minimize variability in coagulation parameters in the *in vitro* model [17,18]. Inclusion/exclusion criteria are described in Supplementary Table S2. Blood was collected in trisodium citrate and used immediately for ROTEM (Werfen) or was centrifuged twice at 2500 × *g* for 15 minutes and snap-frozen in liquid nitrogen before storage at −80 °C for later fibrin formation assays.

To mimic TIC, whole blood or plasma was incubated with the following mediators of coagulation and fibrinolysis pathways: 1 nM aPC (Thermo Fisher Scientific), 10 μg/mL PS (Bio-Techne), 100 IU/mL tissue plasminogen activator (Actilyse; Boehringer Ingelheim), and 60 mU/mL plasmin (Sigma Aldrich). Concentrations were chosen based on dose-response experiments (Supplementary Figure S1). In the whole blood TIC model, TIC components were combined with 50% hemodilution. A dose-response study was performed, showing that 50% dilution in saline (0.9% NaCl) impaired clot formation as assessed by ROTEM (Supplementary Figure S2).

Whole blood and plasma samples were then supplemented with 22.7 μg/mL FV (1 U/mL), 22.7 μg/mL FVa (Haemtech), 0.2 mg/mL TXA (Mylan), 0.8 mg/mL fibrinogen concentrate (Fibryga; Octapharma), or 0.5 U/mL PCC (Cofact; Sanquin). Based on a hypothetical distribution volume of 5 L, chosen concentrations for these compounds are equal to a dose of 1 g TXA, 4 g fibrinogen, and 2500 U of PCC. The effect of FV(a) and PCC as additional therapies to TXA and fibrinogen was evaluated in separate experiments. The effect of the purified FV and FVa product was confirmed using FV-depleted plasma (Supplementary Figure S3). A dose-response experiment was performed for FV and FVa in the whole blood TIC model.

### 2.3 ROTEM in a whole blood TIC model

ROTEM (Tem International GmbH) EXTEM was performed according to the manufacturer’s guidelines. The EXTEM assay measures the TF-initiated pathway of whole blood coagulation by the addition of recombinant TF, CaCl_2_, and phospholipids. Although the TF concentration in the EXTEM reagents is not provided by the manufacturer, it is estimated to be at least 25 pM [19].

Clotting time (CT) is the time until the clot reaches 2 mm in amplitude. Maximum clot firmness (MCF) represents the maximum amplitude. Maximum lysis (ML) is the percentage of lysis detected during the assay duration (90 minutes). Lysis time represents the time in minutes from CT until ML.

### 2.4 Fibrin formation assay in a plasma TIC model

Citrated plasma was added to a 96-well plate and supplemented with TIC components. Next, single treatments or a combination of treatments were added to the wells. Appropriate vehicles were used to account for the buffer volumes. Coagulation was then initiated by the addition of a coagulation buffer, resulting in a final concentration of 15 mM CaCl_2_, 4 μM phospholipids (1,2-Dioleoyl-sn-glycero-3-phosphoethanolamine:1,2-Dioleoyl-sn-glycero-3-phospho-L-serine:1,2-Dioleoyl-sn-glycero-3-phosphocholine, 50:30:20; Polarlipids), and 1.1 pM or 5.5 pM TF (Innovin; Siemens) [20]. Final volume per well was 70 μL, consisting of 81.25% plasma, 12.5% additives (TIC components and/or therapies), and 6.25% of concentrated coagulation buffer. Absorbance (ie, turbidity) was measured at 405 nm every 15 seconds for 2 hours using the SpectraMax M2 plate reader (Molecular Devices). Outcomes included lag time and maximum optical density (OD).

### 2.5 Statistical analysis

All data were analyzed using SPSS version 25.0 (IBM). Graphs were made using GraphPad Prism version 9.0. Continuous nonparametric variables are presented as median with IQR and analyzed with the Kruskal–Wallis test. Binomial data are presented as numbers with percentages and analyzed with chi-square test.

To assess the association between FV and mortality, a binary logistic regression was made with 28-day mortality as the dependent outcome variable and age, injury severity score (ISS), traumatic brain injury (TBI; defined as an abbreviated injury score head and neck ≥3), lactate, fibrinogen, the volume of crystalloids infused prior to blood withdrawal, and FV activity as covariates. The effect of each covariate is displayed as standardized coefficients (β) and odds ratios with 95% CIs. The overall model significance was assessed using chi-squared test. Nagelkerke *R*^*2*^ was used to describe the proportion of variability in 28-day mortality explained by the model. Missing data were excluded from the analysis (Supplementary Table S3).

For the *in vitro* study, data were analyzed with a Friedman 2-way analysis of variance by ranks with pairwise comparisons adjusted by Bonferroni correction or with Wilcoxon signed-rank test. A *P* value of less than .05 was considered to be statistically significant.

## 3 Results

### 3.1 Decreased FV activity is associated with mortality after trauma

**Figure 1:**
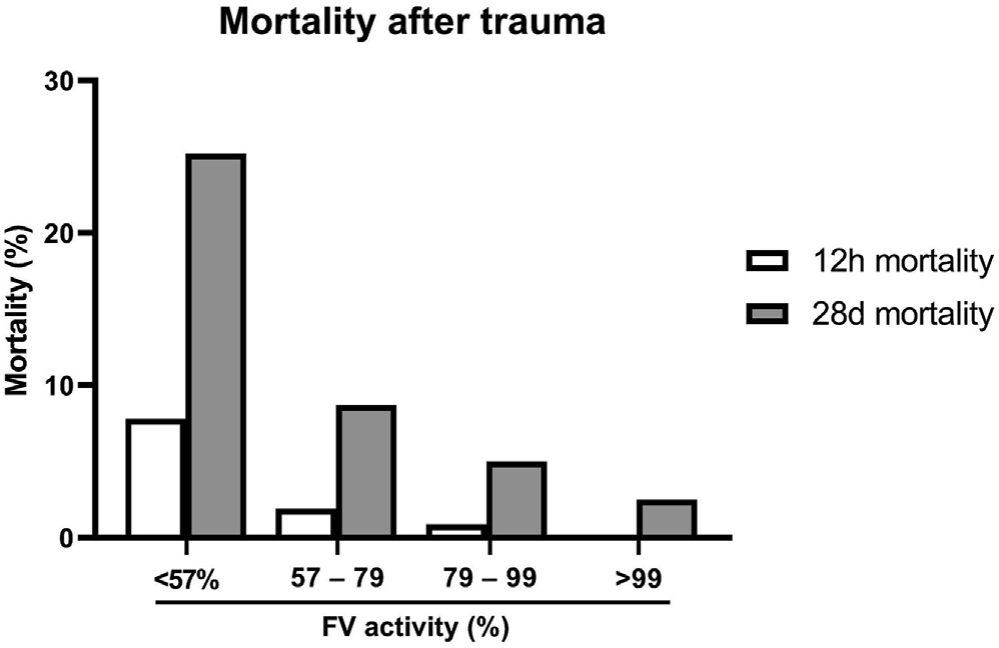
Twelve-hour and 28-day mortality after trauma stratified by factor (F)V activity on emergency room arrival. Quartiles are rounded up for visual clarity. Quartile 1: <56.8%; quartile 2: 56.9% to 79.0%; quartile 3: 79.1% to 99.3%; quartile 4: >99.4%.

A total of 1285 patients were included and divided into quartiles based on FV activity, resulting in the following 4 groups: quartile 1: ≤56.8%; quartile 2: 56.9% to 79.0%; quartile 3: 79.1% to 99.3%; quartile 4: ≥99.4%. Patient characteristics are shown in Table 1. Patients with decreased FV activity were more severely injured and in shock, as shown by an increased ISS and increased lactate concentration compared with the other groups. Decreasing levels of FV activity were associated with increased mortality at both 12 hours and 28 days (Figure 1). Multivariate logistic regression analysis is shown in Table 2. After adjusting for age, ISS, TBI, lactate, fibrinogen, and the volume of crystalloids received prior to blood withdrawal, FV activity remained associated with 28-day mortality (odds ratio, 0.99 [95% CI, 0.98-1.00]; *P* = .009).

**Table 1:** Characteristics of trauma patients.

Characteristics	Whole cohort N = 1285	First quartile n = 321	Second quartile n = 323	Third quartile n = 318	Fourth quartile n = 323
FV activity, % (range)		<56.8	56.9-79.0	79.1-99.3	>99.4
**Demographics**					
Age (y)	39 (26-54)	38 (25-55)	36 (26-55)	39 (26-53)	40 (28-55)
Male	1005 (78)	234 (73)	255 (79)	247 (78)	269 (83)
Blunt injury	971 (84)	240 (89)	239 (82)	247 (84)	245 (80)
TBI	361 (29)	135 (43)	88 (28)	72 (23)	66 (21)
ISS	16 (8-26)	27 (15-38)	17 (8-26)	10 (4-21)	9 (4-17)
Crystalloids prior to blood withdrawal (mL)	0 (0-400)	0 (200-750)	0 (0-500)	0 (0-250)	0 (0-100)
**Shock parameters**					
GCS	14 (10-15)	13 (5-15)	14 (10-15)	15 (12-15)	15 (13-15)
Hb (g/dL)	13.8 (12.6-14.8)	13.0 (11.6-14.3)	13.8 (12.6-14.7)	14.0 (13.0-15.0)	14.2 (13.2-14.9)
Lactate (mmol/L)	2.2 (1.4-3.4)	3.0 (1.8-5.1)	2.0 (1.3-3.5)	2.0 (1.2-2.8)	2.0 (1.3-2.8)
BD (mmol/L)	1.6 (−0.4 to 4.5)	4.7 (−1.5 to 8.8)	1.9 (−0.5 to 4.4)	0.9 (−0.8 to 3.0)	0.7 (−1.1 to 2.4)
**Coagulation**					
INR	1.1 (1.0-1.1)	1.1 (1.0-1.3)	1.1 (1.0-1.1)	1.0 (1.0-1.1)	1.0 (1.0-1.1)
aPTT (s)	25 (23-28)	28 (25-34)	24 (23-27)	24 (22-26)	24 (22-26)
Fibrinogen (g/L)	2.1 (1.6-2.6)	1.4 (1.1-1.8)	1.9 (1.6-2.4)	2.3 (1.9-2.7)	2.4 (2.0-2.9)
Platelet count (×10^9^/L)	225 (186-267)	206 (167-252)	222 (185-264)	230 (198-273)	239 (206-274)
**Transfusion at 12 h**					
Crystalloids (mL)	1000 (100-2200)	2000 (1000-3000)	1000 (200-2600)	1000 (0-1900)	800 (0-1500)
PRBCs (units)	0 (0-2)	2 (0-7)	0 (0-2)	0 (0-0)	0 (0-0)
Plasma (units)	0 (0-0)	0 (0-4)	0 (0-0)	0 (0-0)	0 (0-0)
Platelets (units)	0 (0-0)	0 (0-1)	0 (0-0)	0 (0-0)	0 (0-0)

Data are represented as *n* (%) or median (IQR). Differences between quartiles were analyzed with the Kruskal–Wallis test for continuous variables or the chi-square test for binomial variables.

aPTT, activated partial thromboplastin time; BD, base deficit; FV, factor V; GCS, Glasgow Coma Scale; Hb, hemoglobin; INR, international normalized ratio; ISS, injury severity score; PRBC, packed red blood cell; TBI, traumatic brain injury.

**Table 2:** Univariate and multivariate models on 28-day mortality after trauma.

Characteristics	Univariate model			Multivariate model		
	β	Odds ratio (95% CI)	P value	β	Odds ratio (95% CI)	P value
Age (y)	0.03	1.03 (1.02-1.04)	<.001	0.04	1.04 (1.03-1.05)	<.001
Injury Severity Score	0.08	1.08 (1.07-1.10)	<.001	0.03	1.04 (1.02-1.06)	<.001
TBI	1.63	5.09 (3.70-7.49)	<.001	1.18	3.25 (1.99-5.31)	<.001
Lactate (mmol/L)	0.14	1.15 (1.10-1.20)	<.001	0.10	1.11 (1.04-1.17)	.001
Fibrinogen (g/dL)	−1.62	0.20 (0.14-0.28)	<.001	−0.81	0.45 (0.29-0.69)	<.001
FV (10% change)	−0.40	0.67 (0.62-0.72)	<.001	−0.14	0.87 (0.78-0.97)	.009
Crystalloids prior to blood withdrawal (×100 mL)	−0.11	1.12 (1.08-1.15)	<.001	0.02	1.02 (0.98-1.07)	.36

The model was statistically significant (chi-square = 233; *P* < .001) and Nagelkerke *R*^*2*^ = .39.

FV, factor V; TBI, traumatic brain injury.

### 3.2 The effect of FV(a) supplementation in a whole blood TIC model

**Figure 2:**
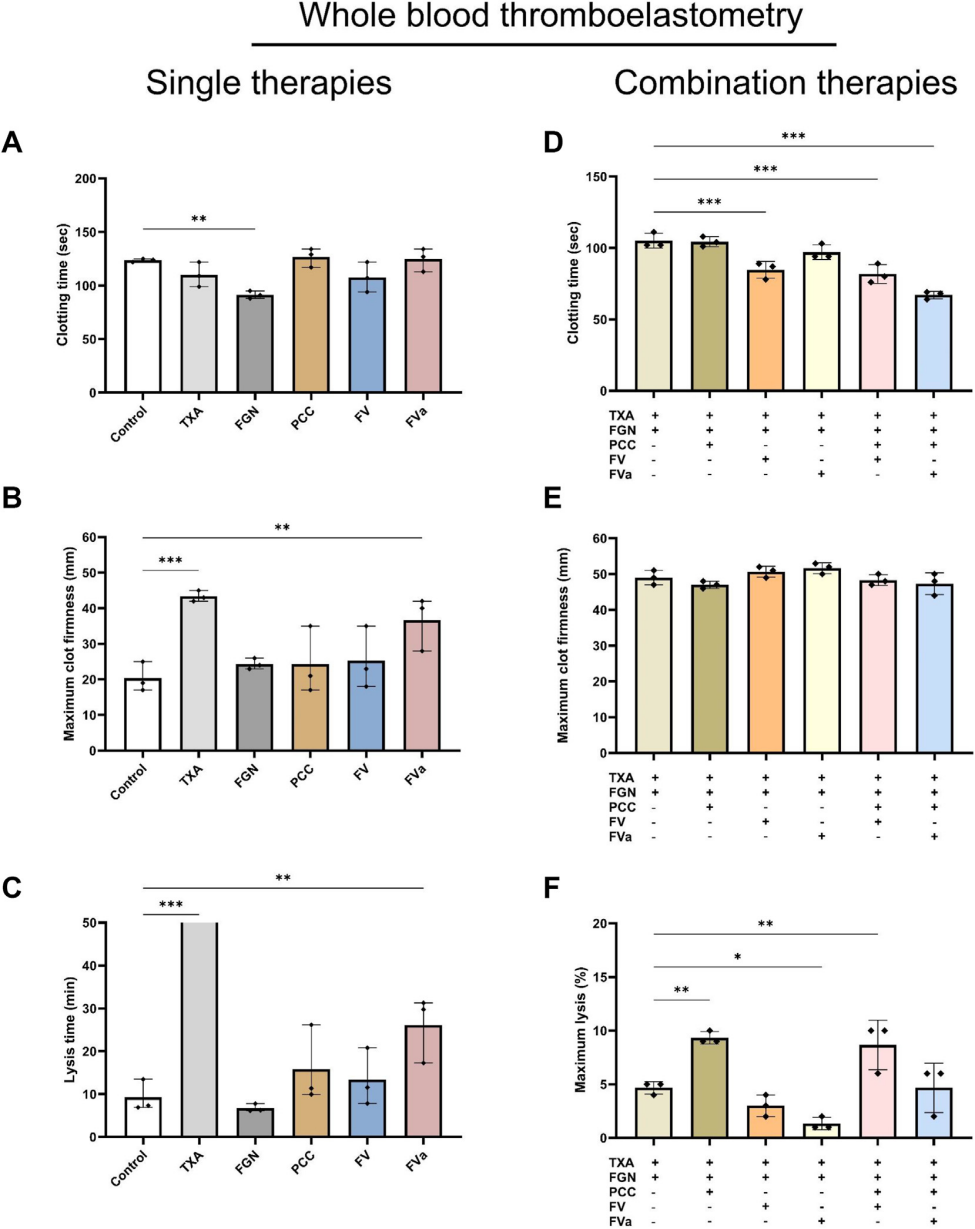
Effects of factor (F)V (1 U/mL of purified coagulation FV) in relation to other treatments on whole blood coagulation. Whole blood from 3 healthy volunteers was 50% diluted in saline and spiked with 1 nM activated protein C, 10 μg/mL protein S, 100 IU/mL tissue plasminogen activator, and 60 mU/mL plasmin. Both single therapies (A–C) and combination therapies (D–F) were added. FGN, 0.8 mg/mL fibrinogen concentrate; FVa, equivalent weight in μg/mL as FV of purified coagulation FVa; PCC, 0.5 U/mL prothrombin complex concentrate; TXA, 0.2 mg/mL tranexamic acid. All data points are shown. ∗*P* < .05, ∗∗*P* < .01, ∗∗∗*P* < .001 compared with the control.

The effect of single treatments on ROTEM parameters is shown in Figure 2A–C. As a single therapy, FV did not show any improvement in deranged EXTEM parameters compared with control. However, FVa significantly increased MCF compared with control. This effect was not seen with a lower dose of FVa (Supplementary Figure S4). Additionally, FVa inhibited fibrinolysis as lysis time was significantly longer. As anticipated, TXA effectively inhibited fibrinolysis, which was associated with a significant increase in MCF. Fibrinogen reduced CT but did not affect MCF. PCC did not improve measures of coagulation.

Given that both TXA and fibrinogen concentrate are widely utilized to treat TIC, we sought to investigate the effects of FV(a) when given in conjunction with these treatments, and we also compared this strategy with the addition of PCC (Figure 2D–F). The addition of FV to TXA and fibrinogen resulted in a significant decrease in CT without affecting MCF or other ROTEM parameters. Addition of FVa did not affect clot build-up but did result in a decreased ML. The addition of PCC to TXA and fibrinogen did not result in a significantly different CT compared with TXA and fibrinogen alone. Interestingly, CT significantly decreased with the addition of FV or FVa to PCC. The combination of PCC with TXA and fibrinogen was associated with increased fibrinolysis compared with TXA and fibrinogen alone.

### 3.3 The effect of FV(a) in a plasma TIC model

**Figure 3:**
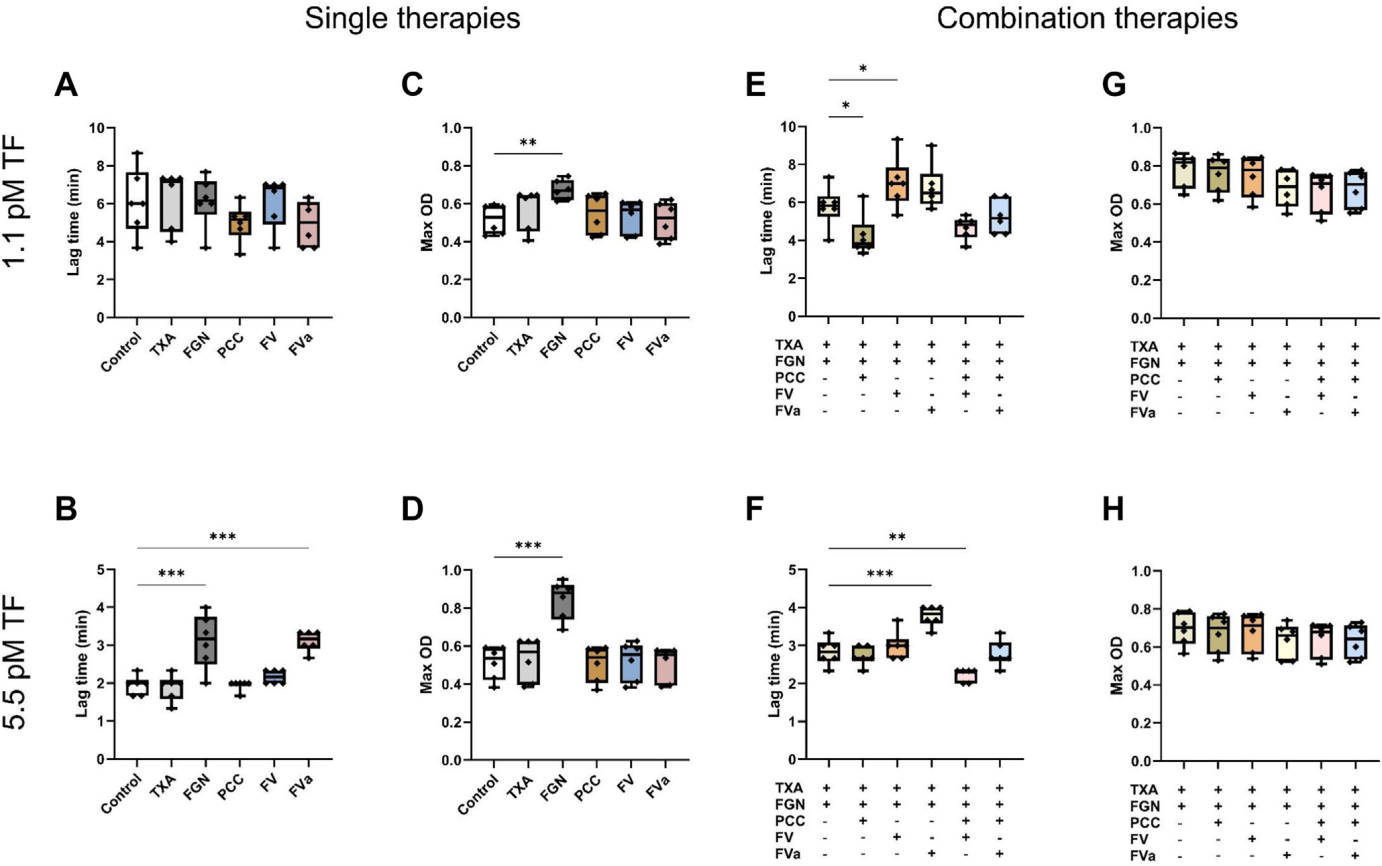
Effects of factor (F)V (1 U/mL purified coagulation FV) in relation to other treatments on fibrin formation. Plasma from 6 healthy volunteers was incubated with 1 nM activated protein C, 10 μg/mL protein S, 100 IU/mL tissue plasminogen activator, and 60 mU/mL plasmin. Both single therapies (A–D) and combination therapies (E–H) were tested in the presence of 15 mM CaCl_2_ and 4 μM phospholipid concentrate with either low (A, C, E, G) or high (B, D, F, H) tissue factor (TF) concentrations as indicated. FGN, 0.8 mg/mL fibrinogen concentrate; FVa, equivalent dose in μg/mL of FV purified coagulation FVa; OD, optical density; PCC, 0.5 U/mL prothrombin complex concentrate; TXA, 0.2 mg/mL tranexamic acid. All data points are shown. ∗*P* < .05, ∗∗*P* < .01, ∗∗∗*P* < .001 compared with control.

The effect of single treatments on fibrin formation is shown in Figure 3A–D. As a single therapy, FV and FVa did not improve fibrin formation compared with control. Contrarily, in whole blood conditions with high TF, FVa resulted in a prolonged lag time compared with control. TXA fully inhibited fibrinolysis with both low and higher amounts of TF. As expected, fibrinogen supplementation resulted in a significantly increased maximum OD with both low and high TF compared with control. With high TF, fibrinogen supplementation prolonged lag time. PCC did not result in statistically significant deviations from the control.

The results from the combined treatments are shown in Figure 3E–H. Similarly, to their single treatment effects, FV and FVa significantly prolonged lag time in combination with TXA and fibrinogen at 2 TF concentrations. The addition of PCC to TXA and fibrinogen reduced lag time at lower TF concentrations. With higher TF concentrations, FV in combination with PCC reduced lag time, whereas other combination treatments did not affect fibrin formation. No differences between combination therapies were seen in maximum OD.

## 4 Discussion

In this study, we showed that decreased FV activity after trauma is associated with increased mortality. In a whole blood model of TIC, FV(a) improved clot build-up and clot strength and decreased fibrinolysis both as a single therapy and when combined with TXA and fibrinogen. In a plasma TIC model, in the presence of much lower amounts of TF compared with the whole blood model, FV(a) delayed fibrin formation as a single therapy, and when combined with TXA and fibrinogen.

Our finding that FV depletion is associated with mortality aligns with previous cohort studies showing diminished FV levels in severely injured patients as well as lower levels in deceased patients compared with patients who survived [10,21,22]. The multivariate regression analysis showed that the association between decreased FV activity and 28-day mortality is (in part) independent of age, injury severity, TBI, shock, volume of crystalloids infused prior to blood withdrawal, and fibrinogen. Unlike other coagulation factors, FV is often reduced to levels less than 50%, which could be explained by several mechanisms. First, FV is proteolyzed by α-thrombin, forming FVa, which is inactivated by aPC [23,24]. Second, plasmin can degrade FV and FVa, as suggested by *in vitro* studies [12,25]. Dilution by infusion of large volumes of crystalloids likely aggravates the decrease in FV activity. It is, however, unknown at which concentration FV becomes too low for adequate thrombin generation. *In vitro* studies suggest that coagulation factor activity of more than 25% to 50% is generally sufficient [26]. For FV activity, this percentage is possibly even lower [27,28]. Of note, 20% of FV is stored in platelet α-granules, which have been shown to be more resistant to aPC degradation. Thereby, the FV activity measured in plasma may not reflect the effective FV activity in a trauma patient [29]. For example, congenital FV deficiency can often manifest itself as no or only mild bleeding tendency, which is thought to be related (at least partly) to platelet-derived FV [30]. These cases, however, reflect isolated deficiencies instead of a combination of coagulation factor deficiencies as seen in TIC.

We aimed to mimic TIC using a model that included increased activation of the aPC and plasmin pathways, as these contribute to TIC and FV depletion [11]. Additionally, hemodilution of whole blood was applied, resulting in dilution of all coagulation factors. In this whole blood TIC model, FV(a) improved coagulation as assessed by ROTEM, as it increased MCF and decreased CT. Additionally, FVa delayed the time to lysis, which may be secondary to increased thrombin formation [31]. Of note, the antifibrinolytic effects remained present when FV(a) was also combined with TXA and fibrinogen. A previous mouse study showed that a congenital FV Leiden mutation is associated with inhibition of fibrinolysis, which is in line with our results [32]. Of note, aPC-resistant FV has been shown to be beneficial in bleeding outcomes in an animal model of severe trauma-induced shock [33]. The antifibrinolytic effect of FV(a) we observed could also be mediated through thrombin-activatable fibrinolysis inhibitor (TAFI), as inhibition of TAFI negated the antifibrinolytic effect of FV *in vitro* [32].

Interestingly, we observed differential effects in the plasma-based assay, in which FV did not improve coagulation. Two TF concentrations were used in this assay, which have been shown to correlate with tissue injury severity *in vivo* [10,34]. At a relatively low TF concentration, FV increased CTs when combined with TXA and fibrinogen. At higher TF concentrations, FV did not show any effect, whereas FV(a) was associated with an increased lag time in both single therapy and when combined with TXA and fibrinogen, ie, with longer time until fibrin formation.

The contrasting results between the whole blood and the plasma assays can have several explanations. First, in the ROTEM assay, even higher TF concentrations are used (>3 times higher compared with the fibrin formation assay), which may have depleted FV more rapidly compared with the plasma TIC model. Second, as we incorporated hemodilution in the whole blood model, FV activity was at least as low as 50%, which could explain the beneficial effects of FV(a) under these conditions. In the plasma assay, FV(a) may amplify the anticoagulant response by acting as a cofactor in the aPC pathway for the inactivation of FVa and FVIIIa, as well as through association with TF pathway inhibitor [35]. Taken together, FV appears beneficial in a situation of strong TF-induced activation of coagulation.

Regarding the effect of other therapies in our TIC model, fibrinogen decreased CT in the whole blood model. In the plasma model, however, it was associated with prolonged lag time but increased OD (ie, improved clot strength). Similar to FV(a), the efficacy of fibrinogen may depend on the severity of hyperfibrinogenemia and the TF amount, or in other words, on the severity of the trauma-induced host response. Moreover, fibrinogen may become an anticoagulant under some conditions [36]. An anticoagulant effect of fibrinogen could potentially also explain the lack of benefit of empiric fibrinogen supplementation in trauma patients [7]. In patients with penetrating injury, fibrinogen significantly increased mortality, which can be speculated to be due to an anticoagulant response in conditions of low TF exposure [7].

We observed that PCC was associated with a doubling of the ML when combined with TXA and fibrinogen compared with TXA and fibrinogen alone. In the plasma model, however, PCC was associated with faster clot build-up compared with control, but only at the lowest TF concentration. It can be speculated that the protein C and PS present in PCC increase fibrinolysis through inhibition of plasminogen activator inhibitor 1 or indirectly through TAFI activation, especially under conditions with large amounts of thrombin present, which is a prerequisite for both protein C and TAFI activation [37]. Interestingly, when PCC was combined with TXA and fibrinogen, we observed that the addition of FV(a) was associated with decreased CT in the whole blood TIC model. This is in line with earlier *in vitro* studies, which showed benefits of FV and PCC in the context of direct oral anticoagulant reversal [38].

Taken together, our study illustrates the difficulty in coagulation factor supplementation for TIC, as the outcome depends on many different variables, even within the controlled context of an experimental *in vitro* model. Therefore, empiric treatment of traumatic bleeding with coagulation factors is likely to be an inadequate approach. Our results suggest that in the most severely injured patients (ie, those with high TF expression, depleted FV activity, and hyperfibrinolysis), FV suppletion may prove beneficial, although currently, FV concentrate is not an approved drug. Of note, such patients can potentially be identified based on their ROTEM coagulation profile [8].

This study has several limitations. Within the trauma cohort, there was a transition toward the empiric use of TXA over the course of inclusion, but only a low number of patients received this treatment, which could potentially influence the coagulation factor results [39]. Therefore, whether the relation between FV activity and mortality would be present had TXA been given is not known. Although the observed association between FV activity and mortality was corrected for some important confounding factors, there likely remain additional variables that were unaccounted for in the statistical model. Also, we did not have data on the ethnicity of trauma patients, so differences in FV activity among different ethnic groups could not be assessed. Also, female patients are known to have a different coagulation response following trauma [17,18]. In the *in vitro* model, we only used blood from healthy male volunteers, limiting translation to female patients. We can only speculate about the effect of TIC components on FV activity in the *in vitro* model since the depletion of FV happens during coagulation, at which point it becomes impossible to collect plasma for FV activity measurement. Lastly, we acknowledge that not all components of TIC have been captured in our *in vitro* model, which lacks the spatial heterogeneity of the *in vivo* coagulation system with activated endothelium and blood flow, which should be the focus of future studies [40,41].

In conclusion, FV depletion is associated with increased mortality in trauma patients. The addition of FV(a) in an *in vitro* model of TIC has procoagulant, antifibrinolytic effects and anticoagulant effects, depending on specific assay characteristics such as the amount of TF present. Whether a subgroup of TIC patients exists that would benefit from FV(a) supplementation requires further study.
