# Supplementary material for: The role of factor V in trauma-induced coagulopathy: an observational and experimental study
Source: Res Pract Thromb Haemost. 2025 Apr 17;9(4):102857. doi: 10.1016/j.rpth.2025.102857 (PMC12150085; doi:10.1016/j.rpth.2025.102857)
Supplement: Supplementary Material [file mmc1.docx]

**Supplemental Table S1: In-/exclusion criteria ACIT-III clinical study**

| Inclusion criteria |
| --- |
| Adult trauma patients were enrolled if they sustained a blunt or penetrating trauma and for whom the trauma team was activated, with at least one of the following clinical parameters:   - Respiratory rate < 10 or > 25 times per minute - Heart rate ≥120 beats per minute - Systolic blood pressure < 90 mmHg - Oxygen saturation < 90% - Estimated blood loss ≥ 500 ml - Glasgow Coma Score ≤ 13 or abnormal pupil size and/or reaction   Or clinical signs of at least one of the following diagnoses:   - Femur fracture - Signs of flail thorax/pneumothorax/haematothorax or multiple rib fractures - Signs of significant abdominal injury - Pelvic fracture - Spine injury |
| Exclusion criteria |
| - Age < 18 years old - Patients transferred from other hospitals - Patients presenting more than 120 min after time of injury - Patients who have received more than 2000 ml of intravenous fluids prior to emergency department arrival - Patients with burns > 5% of their body surface area - Patients taking anticoagulant medication other than aspirin (< 650 mg/day) - Patients with a known bleeding diathesis - Patients with moderate to severe liver disease (Child-Pugh B or C3) |

**Supplemental Table S2: In-/exclusion criteria volunteer study**

| Inclusion criteria |
| --- |
| - Males, age: 18-35 years old |
| Exclusion criteria |
| - Participation in a scientific intervention study in the last 3 months - No informed consent - History of coagulation disorders - Active use of prescription medication - Use of anticoagulant medication, including aspirin - History of liver disease - History of chronic transmittable disease - History of alcohol, smoking or drug abuse |

**Supplemental Table S3. Missing data in the logistic regression model**

|  | Data missing, n (%) |
| --- | --- |
| *Age (years)* | 0 (0) |
| *Injury Severity Score* | 14 (1) |
| *Traumatic brain injury* | 42 (3) |
| *Lactate (mmol/l)* | 79 (6) |
| *Fibrinogen (g/dl)* | 23 (2) |
| *FV (10% change)* | 0 (0) |
| *Crystalloids prior to blood withdrawal (x100ml)* | 19 (2) |

**
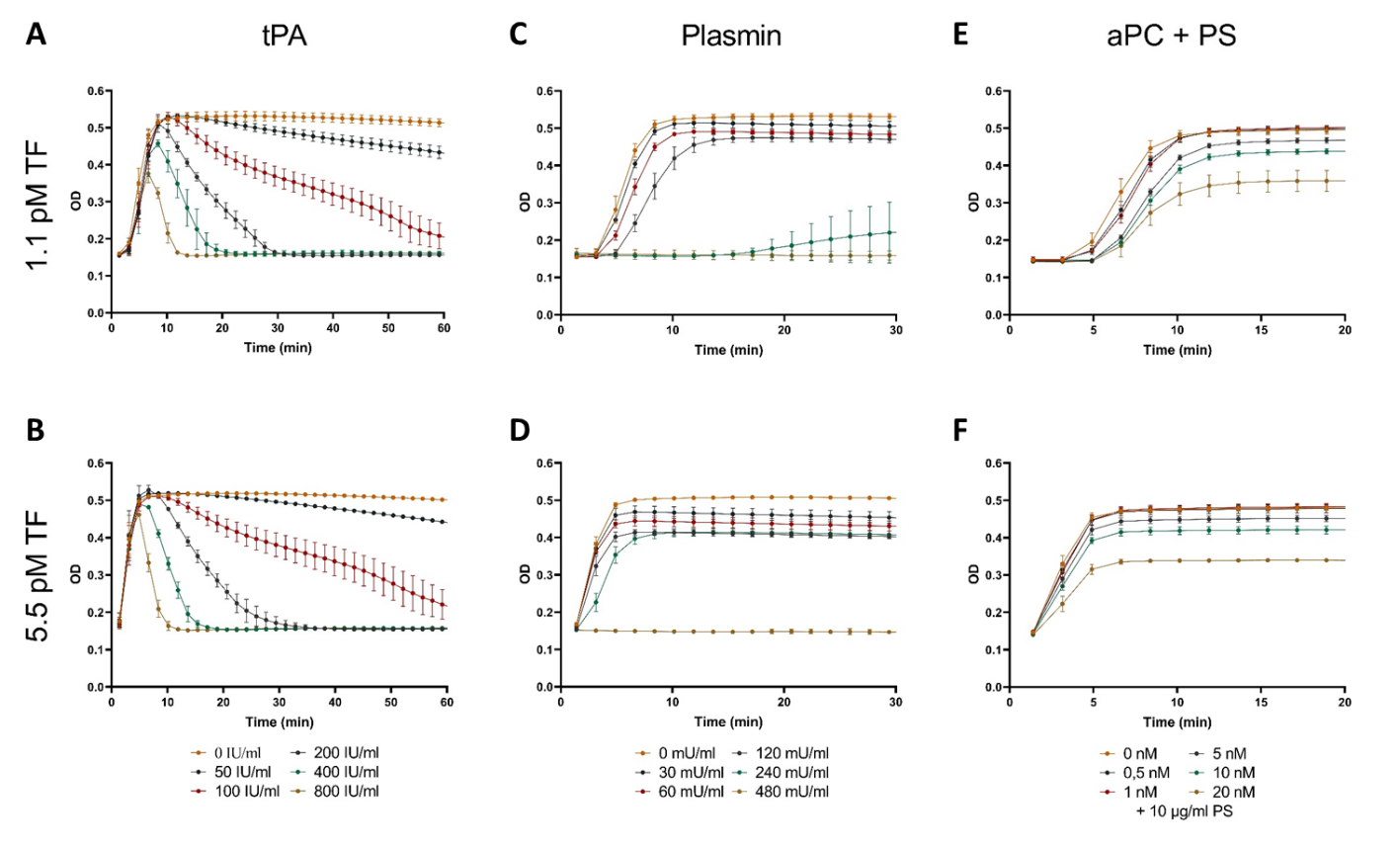
**

**Supplemental Figure S1: *In vitro* effect of components of trauma-induced coagulopathy on fibrin formation.** Fibrin formation was initiated by the addition of 15 mM CaCl_2_, 4 µM phospholipids and 1.1 pM or 5.5 pM tissue factor (final volumes). Optical density (i.e. absorbance) was measured using a spectrophotometer. **A, B)** Tissue plasminogen activator (tPA) **C,D)** Plasmin **E,F)** Activated protein C (aPC) and protein S (PS). Data presented as mean with standard deviation.

**
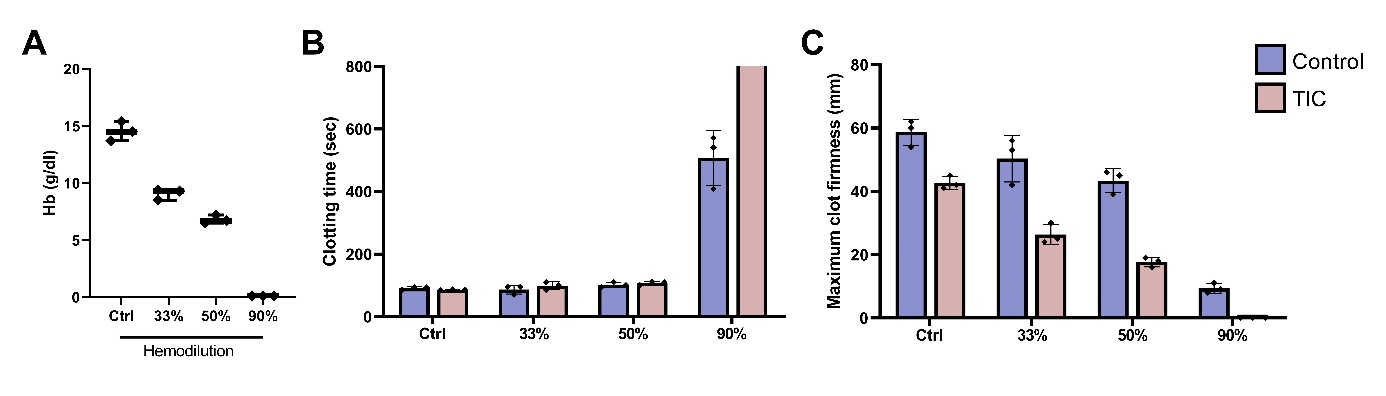
**

**Supplemental Figure S2: *In vitro* effect of haemodilution combined with trauma-induced coagulopathy (TIC) components in rotational thromboelastometry (ROTEM). A)** the effect of diluting whole blood from healthy volunteers with saline (0.9% NaCl) on haemoglobin (Hb). **B)** the effect of haemodilution with or without TIC on clotting time and **C)** maximum clot firmness. TIC components consisted of 100 IU/ml tissue plasminogen activator, 60 mU/ml plasmin, 1 nM activated protein C and 10 µg/ml protein S. Data presented as mean with standard deviation. All data points are shown.

**
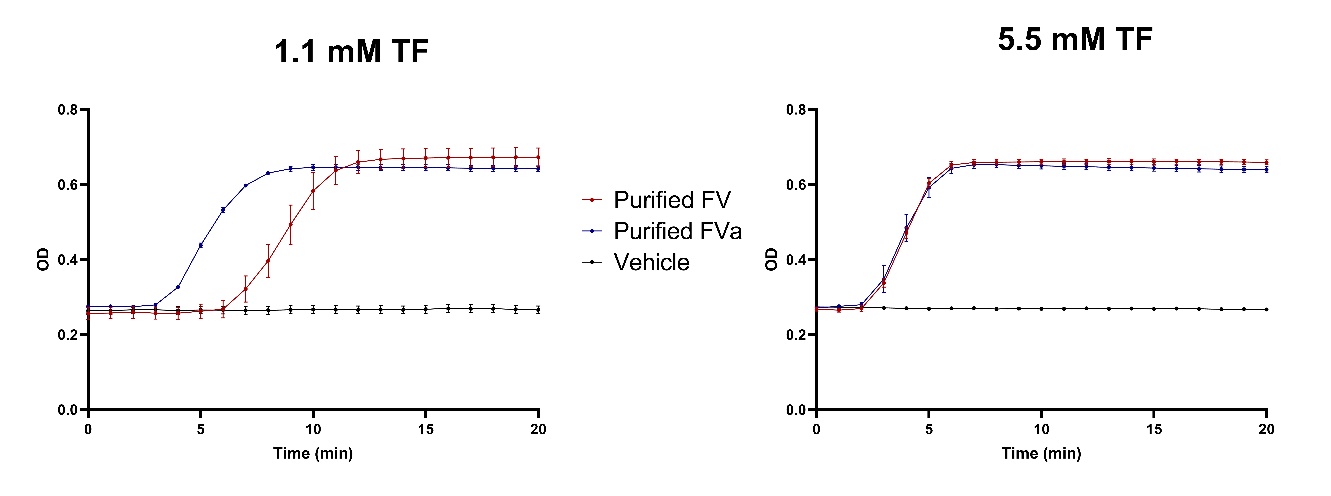
Supplemental Figure S3: *The effect of purified FV and FVa on fibrin formation in FV-deficient plasma.*** *Fibrin formation was initiated with 1.1 mM or 5.5 mM of tissue factor.*


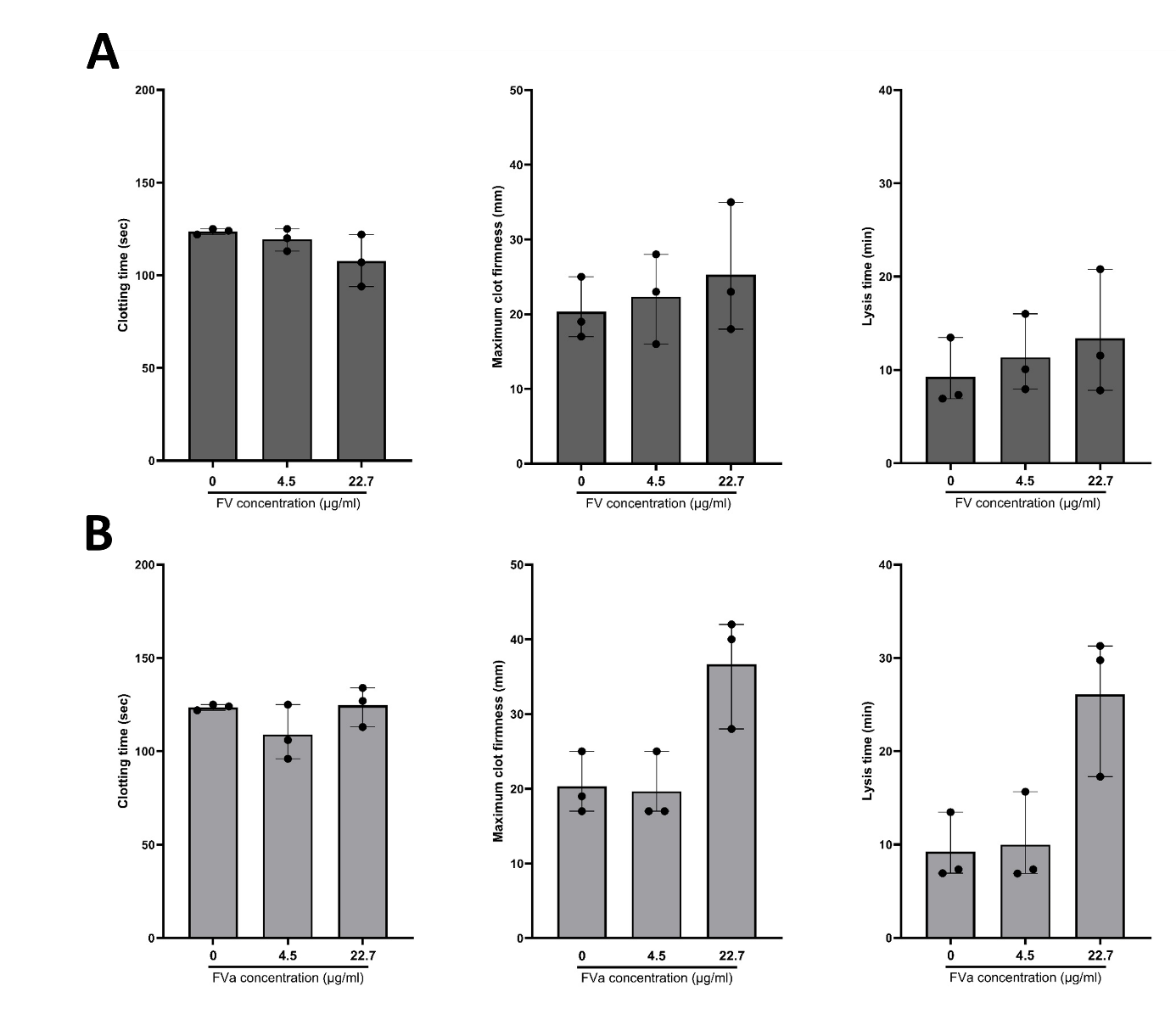


**Supplemental Figure S4: *Dose-response relationship of FV and FVa on rotational thromboelastometry.*** *For both FV (****A****) and FVa (****B****) concentrations of 0, 4.5 and 22.7 µg/ml are displayed.*
